# Hepatitis C Virus Controls Interferon Production through PKR Activation

**DOI:** 10.1371/journal.pone.0010575

**Published:** 2010-05-11

**Authors:** Noëlla Arnaud, Stéphanie Dabo, Patrick Maillard, Agata Budkowska, Katerina I. Kalliampakou, Penelope Mavromara, Dominique Garcin, Jacques Hugon, Anne Gatignol, Daisuke Akazawa, Takaji Wakita, Eliane F. Meurs

**Affiliations:** 1 Institut Pasteur, Unité Hépacivirus et Immunité Innée, Paris, France; 2 Hellenic Pasteur Institute, Molecular Virology Laboratory, Athens, Greece; 3 University of Geneva School of Medicine, Geneva, Switzerland; 4 Institut du Fer à Moulin, Inserm UMRS 839, Paris, France; 5 Lady Davis Institute for Medical Research, Virus-Cell Interactions, McGill University, Montréal, Canada; 6 National Institute of Infectious Diseases, Department of Virology II, Tokyo, Japan; Pohang University of Science and Technology, Republic of Korea

## Abstract

Hepatitis C virus is a poor inducer of interferon (IFN), although its structured viral RNA can bind the RNA helicase RIG-I, and activate the IFN-induction pathway. Low IFN induction has been attributed to HCV NS3/4A protease-mediated cleavage of the mitochondria-adapter MAVS. Here, we have investigated the early events of IFN induction upon HCV infection, using the cell-cultured HCV JFH1 strain and the new HCV-permissive hepatoma-derived Huh7.25.CD81 cell subclone. These cells depend on ectopic expression of the RIG-I ubiquitinating enzyme TRIM25 to induce IFN through the RIG-I/MAVS pathway. We observed induction of IFN during the first 12 hrs of HCV infection, after which a decline occurred which was more abrupt at the protein than at the RNA level, revealing a novel HCV-mediated control of IFN induction at the level of translation. The cellular protein kinase PKR is an important regulator of translation, through the phosphorylation of its substrate the eIF2α initiation factor. A comparison of the expression of luciferase placed under the control of an eIF2α-dependent (IRES^EMCV^) or independent (IRES^HCV^) RNA showed a specific HCV-mediated inhibition of eIF2α-dependent translation. We demonstrated that HCV infection triggers the phosphorylation of both PKR and eIF2α at 12 and 15 hrs post-infection. PKR silencing, as well as treatment with PKR pharmacological inhibitors, restored IFN induction in JFH1-infected cells, at least until 18 hrs post-infection, at which time a decrease in IFN expression could be attributed to NS3/4A-mediated MAVS cleavage. Importantly, both PKR silencing and PKR inhibitors led to inhibition of HCV yields in cells that express functional RIG-I/MAVS. In conclusion, here we provide the first evidence that HCV uses PKR to restrain its ability to induce IFN through the RIG-I/MAVS pathway. This opens up new possibilities to assay PKR chemical inhibitors for their potential to boost innate immunity in HCV infection.

## Introduction

In response to invasion with bacterial or viral pathogens, cells are able to mount an immediate immune response through their ability to use specialized cellular molecules, referred to as pattern recognition receptors or PRRs, to detect unusual DNA, ssRNA or dsRNA structures. Among these PRRs, are the CARD-containing DexD/H RNA helicases RIG-I and MDA5, which are activated upon binding either to both 5′-triphosphate ssRNA and short double-stranded RNA structures (RIG-I) or to long dsRNA and higher-ordered RNA structures (MDA5) [1]. Once activated, these RNA helicases interact with the mitochondria-bound MAVS adapter protein [2]. In the case of RIG-I, the interaction with MAVS requires ubiquitination of the CARD domain of RIG-I by the TRIM25 ubiquitin ligase [3]. Subsequently, MAVS is able to recruit the IKK complex and the TBK1/IKKε kinases that are responsible for the activation of the NF-κB and IRF3/IRF7 transcription factors, respectively. This leads to induction of the interferons and pro-inflammatory cytokines that are involved in the innate immune response [4].

Hepatitis C virus (HCV) (*Hepacivirus* genus within the *Flaviviridae* family) is one of the RIG-I-activating viruses [4], because of its 5′ppp-structured RNA, 3′-structured genomic RNA and replicative RNA duplexes [5]. In particular, its 3′-end poly-U/UC motif has been shown to function in conjunction with the 5′ppp as the HCV structure that activates RIG-I [6].

In contrast to other RIG-I activating viruses such as Sendai virus, influenza, or vesicular stomatitis virus [1], HCV is a poor inducer of IFN and pro-inflammatory cytokines in cell culture systems. One reason for this is that the HCV NS3/4A protease cleaves MAVS, resulting in a rapid disruption of the function of MAVS and in abrogation of the IFN induction pathway [2]. However, data presented in some studies performed using Huh7 hepatoma cells infected with cell-culture generated JFH1 virus showed that the IFN pathway was poorly activated even before full cleavage of MAVS, since only limited nuclear translocation of IRF3 could be detected [7], [8]. Similarly, in another study, infection of Huh7 cells with JFH1 did not lead to any IFN induction, whereas the cells responded well to transfection by synthetic dsRNA poly(I)-poly(C) [9]. Thus, the early events leading to IFN induction after RIG-I activation by HCV are still not well-characterized.

Here, we have re-addressed the question as to whether HCV infection can activate RIG-I/MAVS before cleavage of MAVS by the NS3/4A protease, by performing kinetics of infection with JFH1 in the newly described JFH1-permissive Huh7.25/CD81 cells, which were manipulated to present a functional RIG-I/MAVS pathway. Our results show that HCV infection can stimulate the IFN induction pathway up to 12 hrs post-infection, whereas detection of MAVS cleavage begins at 18 hrs post-infection and is maximal at 24 hrs. Importantly, our data reveal that 12 hrs post-infection, HCV promotes a rapid inhibition of IFN induction at the level of translation, indicating a new mechanism of regulation. We demonstrated that this regulation was linked to activation of the dsRNA-activated eIF2α kinase PKR [10]. Altogether, our results show that HCV uses PKR to control the translation of newly transcribed IFN mRNAs before sufficient NS3/4A protein can be synthesized to efficiently restrain transcription of IFN.

## Results

### Ectopic expression of TRIM25 allows IFN induction in JFH1 permissive Huh7.25/CD81 cells to be studied

**Figure 1:**
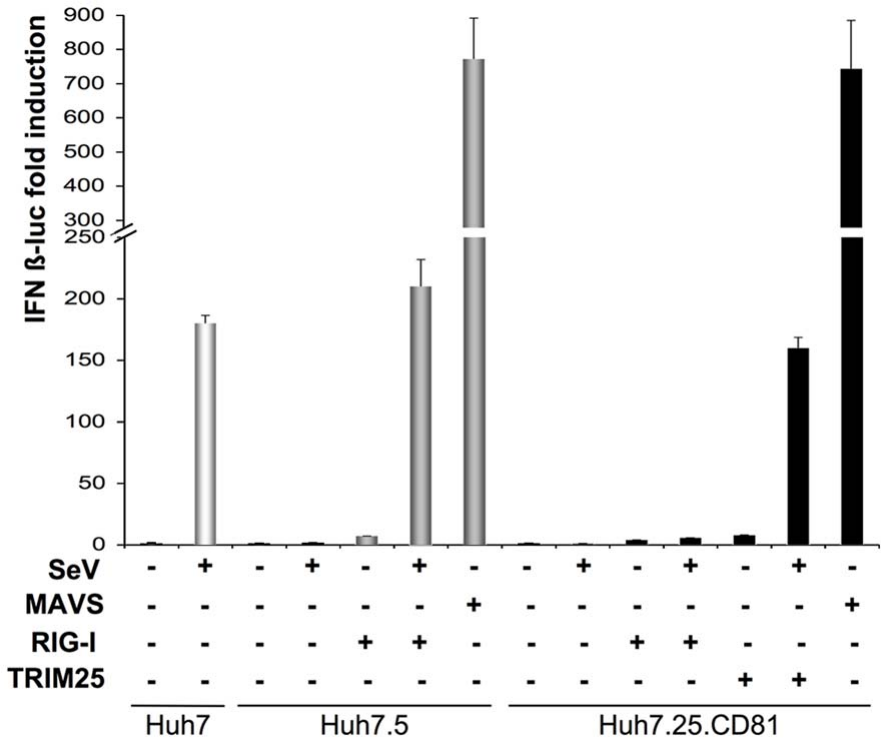
Ectopic expression of TRIM25 restores IFN induction in Huh7.25.CD81 cells. Huh7, Huh7.5 and Huh7.25.CD81 cells were transfected with the pGL2-IFNβ-FLUC/pRL-TK-RLUC reporter plasmids alone or the in presence of plasmids expressing RIG-I (200 ng), TRIM25 (100 ng) or MAVS (400 ng). 24 hrs post transfection, cells were mock-infected or infected with Sendaï virus (SeV) (40 HAU/ml). 24 hrs after infection, luciferase activity was measured and F-luc was normalized against R-luc. IFN expression was expressed as fold induction over control cells that were simply transfected with pGL2-IFNβ-FLUC/pRL-TK-RLUC. Error bars represent the mean ± S.D. for triplicates.

There is at present no satisfactory cell culture system that is both permissive for HCV and presents an intact RIG-I pathway. For instance, the Huh7.5 cells, which were cloned from the hepatoma Huh7 cells for their efficacy to support HCV replication [11], are unable to stimulate IFN induction because of a T55I substitution in the first CARD domain of RIG-I that prevents the association of RIG-I with MAVS [5]. Recently, another Huh7 subclone that could efficiently replicate the JFH1 subgenomic replicon (clone Huh7.25) was stably transfected with the essential HCV CD81 receptor, to generate Huh7.25/CD81 cells that are highly permissive to infection by JFH1[12]. We compared the ability of Huh7.25/CD81, Huh7.5 and Huh7 cells to activate the IFN inducing pathway after transfection with an IFNβ-luciferase reporter and infection with Sendai virus (Figure 1). The results showed that Huh7.25/CD81 cells are defective in the IFN-inducing pathway, similarly to Huh7.5 cells. In contrast, Huh7 cells can mount a robust IFN induction in response to infection with Sendai virus. Interestingly, whereas ectopic expression of MAVS in both Huh7.25/CD81 and Huh7.5 cells resulted in a strong stimulation of IFNβ-luciferase, ectopic expression of RIG-I was not able to restore the IFN-inducing pathway in Huh7.25/CD81 cells, in contrast to in Huh7.5 cells where it was able to do so as expected. This indicated that the defect in Huh7.25/CD81 cells was upstream of MAVS and either at the level of RIG-I or downstream of RIG-I. Since efficient activation of MAVS by RIG-I has been shown to depend upon the ubiquitination of RIG-I by the ubiquitin ligase TRIM25 [3], we assayed the effect of TRIM25 in Huh7.25/CD81 cells. Ectopic expression of TRIM25 in Huh7.25/CD81 cells had no effect alone, but resulted in a strong induction of IFN when the cells were infected with Sendai virus, indicating that these cells present a defect in the RIG-I/MAVS pathway at the level of TRIM25. Whatever the nature of this defect, ectopic expression of TRIM25 now allows the use of Huh7.25/CD81 cells as a model to examine the effect of HCV infection on the IFN induction pathway.

### HCV specifically stimulates the IFN induction pathway during the first 12 hrs of infection, but inhibits it thereafter

**Figure 2:**
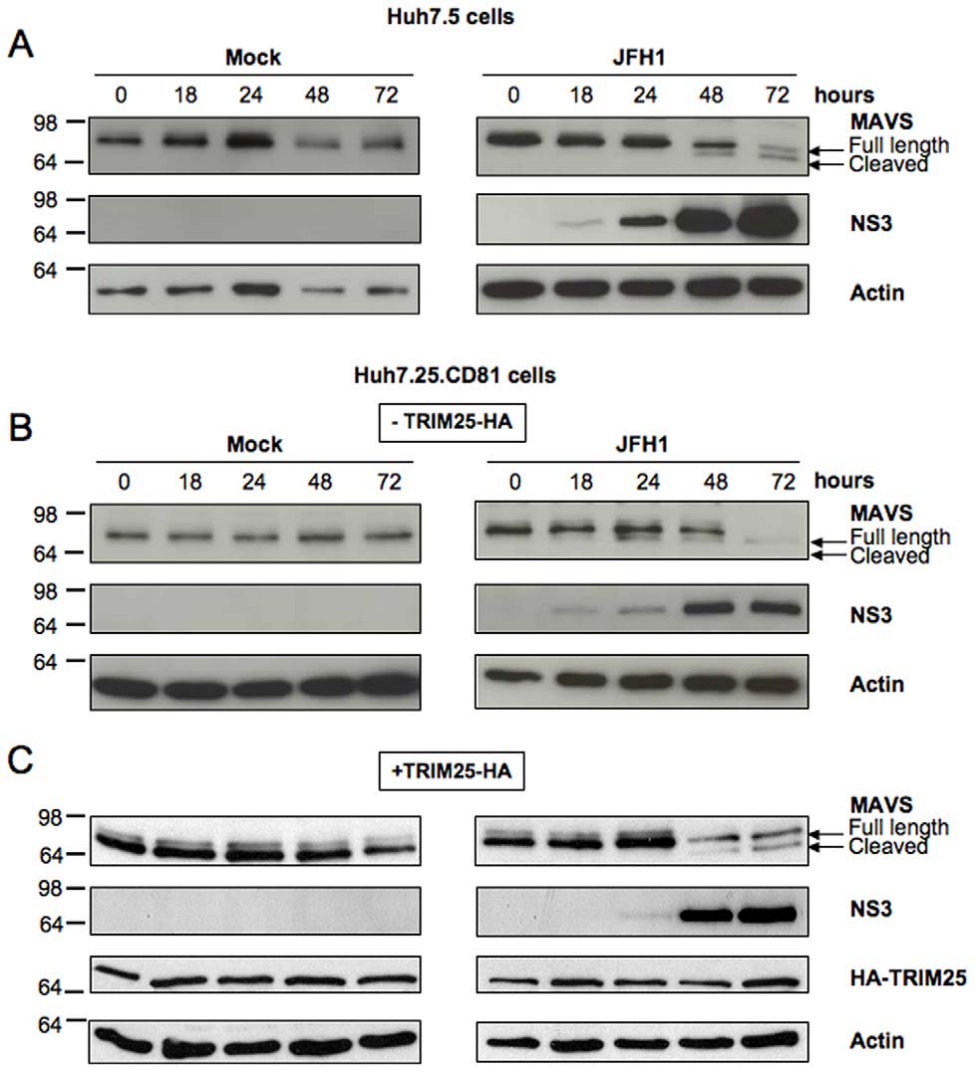
Kinetics of MAVS cleavage in Huh7.25.CD81 cells after JFH1 infection. Huh7.5 cells and Huh7.25.CD81 cells were transfected with an HA-TRIM25 expressing plasmid or with an empty plasmid. 24 hrs post-transfection, cells were mock-infected or infected with JFH1 (m.o.i = 0.05) for the indicated times and cell lysates were generated. Cell extracts (50 µg) were subjected to SDS-12.5% PAGE and blotted with anti-MAVS, anti-NS3, anti-HA or anti-actin as indicated. The arrows indicate the position of full-length MAVS and MAVS cleaved in the presence of HCV NS3/4A.

To estimate the time-frame required to study IFN induction before its abrogation upon the action of NS3/4A, we first established the kinetics of MAVS cleavage in the two HCV permissive cell lines Huh7.25/CD81 and Huh7.5 following HCV infection. MAVS cleavage can be detected by immunoblotting, which shows a progressive diminution of the MAVS full-length protein (540 aa) and the apparition of a MAVS cleavage product (513 aa) [2]. In our experimental conditions, MAVS cleavage was clearly detected in both cell types at 48 hrs after infection with JFH1 (Figures 2A and B). Since ectopic expression of TRIM25 in Huh7.25/CD81 cells will be needed in our subsequent experiments designed to examine the effects of HCV on the IFN induction pathway, we also established the kinetics of MAVS cleavage in these cells after their transfection with a plasmid that expresses HA-TRIM25. Under these conditions, MAVS cleavage was clearly observed at 48 hrs (Figure 2C). In all cases, the viral NS3 protease was detected as early as 18–24 hrs, and thereafter its expression increased strongly with time, concomitantly with MAVS cleavage.

**Figure 3:**
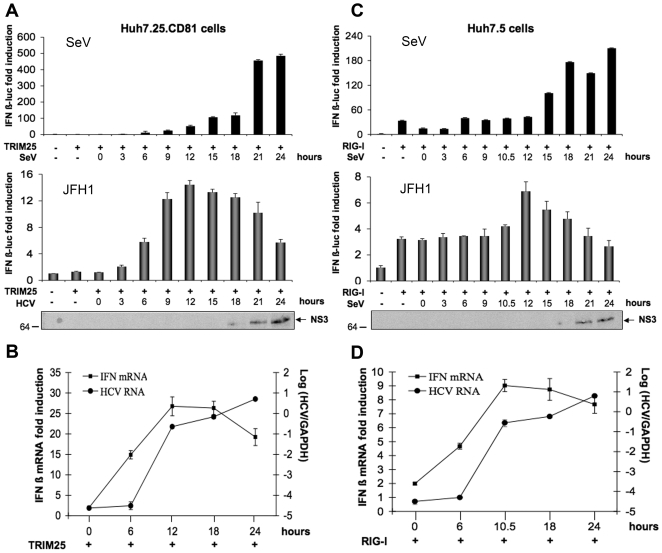
HCV induces IFN during the first 12 hrs of infection and inhibits it thereafter. Huh7.25.CD81 and Huh7.5 cells were transfected with the pGL2-IFNβ-FLUC/pRL-TK-RLUC reporter plasmids together with plasmids expressing HA-TRIM25 (Huh7.25.CD81; A and B) or RIG-I (Huh7.5; C and D). 24 h post-transfection, the cells were infected with SeV (40 HAU/ml) or JFH1 (m.o.i = 0.2). **A and C**: 24 hrs post-transfection, the cells were infected with SeV (40 HAU/ml) or JFH1 (m.o.i = 0.2). At the times indicated, cell lysates were prepared and analysed for IFN induction as described in Materials and Methods. The graphs represent the levels of F-luc activity normalized to R-luc RNA expressed as IFN-β fold-induction over control cells that were simply transfected with pGL2-IFNβ-FLUC/pRL-TK-RLUC. Error bars represent the mean ± S.D. for triplicates. In addition, cell lysates from JFH1-infected cells were pooled and analysed for the presence of NS3 as a marker of HCV infection. **B and D**: 24 hrs post-transfection, Huh7.25.CD81 and Huh7.5 cells were infected with JFH1 (m.o.i = 0.2). At the times indicated, cells were processed for RNA extraction and HCV or IFNβ RNA were quantified by qRT-PCR respectively, and normalized against RNA from GAPDH. Error bars represent the mean ± S.D. for triplicates.

Huh7.25/CD81 or Huh7.5 cells were transfected with an IFNβ-luciferase reporter plasmid in the presence of limited amounts of TRIM25 or RIG-I, respectively, and infected with HCV at an moi of 0.2 or with Sendai virus as control. In case of HCV infection, analysis of luciferase activity or of the expression of endogenous IFNβ RNA and HCV RNA was performed in parallel. The results show an increase in luciferase activity that corresponds to an increase in the activation of the RIG/MAVS pathway (Figure 3A,C bottom). This increase was detected as early as 6 hrs after infection for Huh7.25/CD81 cells and 10.5 hrs for Huh7.5 cells, with a maximum expression at 12 hrs for both cell types. The data of the reporter assay were also confirmed at the endogenous level when induction of endogenous IFNβ RNA was assessed by RTqPCR (Figure 3B,D). In both cases, the expression of the viral HCV RNA increases from 6 hrs after infection and continues to rise thereafter. These results indicate that HCV can activate the RIG-I signalling pathway with concomitant IFN induction within the first 12 hrs of infection.

Intriguingly, we observed that after 12 hrs of infection, there was a considerable decline of luciferase activity in both cell types, whereas the levels of endogenous IFNβ RNA remained at a plateau level until 18 hrs, before decreasing at 24 hrs post-infection. The decrease in luciferase expression between 12 and 18 hrs was surprising since NS3 was not fully expressed within this period of time (Figure 3A,C). In contrast, a decrease in luciferase expression at 24 hrs was expected, due to the massive proteolytic cleavage of MAVS by the HCV NS3/4A protease (Figure 2). Inhibition of luciferase activity was shown to be specific to HCV, as infection of both cell types with Sendai virus resulted in a continuous increase of luciferase activity from the IFNβ-luc reporter plasmid (Figure 3A,C top). Altogether, our data indicate that HCV can activate the IFN induction pathway within 12 hrs after infection, but blocks it prior to the NS3/4A-mediated cleavage of MAVS.

### HCV activates the phosphorylation of PKR and of its substrate eIF2α, as of 12 hrs post-infection

**Figure 4:**
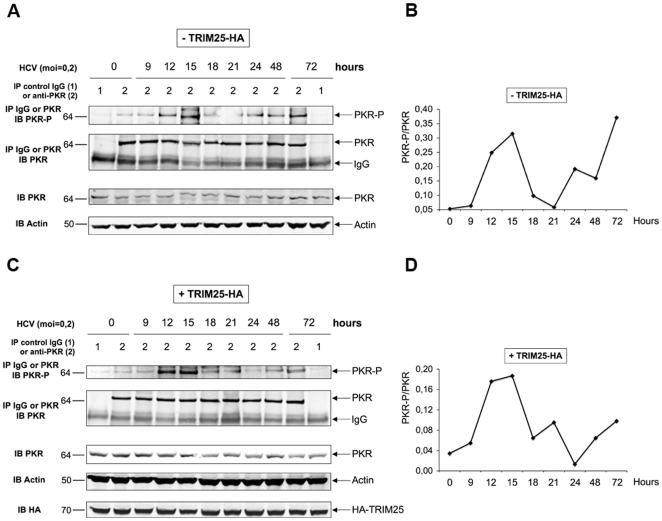
HCV activates the phosphorylation of PKR at 12 and 15 hrs post infection. **A and C**: Huh7.25.CD81 cells, plated into 100 cm^2^ plates, were transfected with the HA-TRIM25 expressing plasmid or with an empty plasmid. 24 hrs post-transfection, cells were infected with JFH1 at an m.o.i of 0.2. At the indicated times post-infection, cell extracts (1 mg) were incubated with Mab 71/10 anti-PKR. In addition, cell extracts prepared at time 0 or at 72 hrs p.i. were incubated with mouse IgG as a control of specificity. The immunoprecipitated complexes were run on two different NuPAGE gels and blotted using Mab 71/10 or anti-phosphorylated PKR (PKR-P). The presence of PKR and PKR-P was revealed using the Odyssey procedure. **B and D**: The bands corresponding to total PKR and their corresponding phosphorylated proteins were quantified using the Odyssey software and expressed as the ratio PKR-P/PKR in the absence (B) or presence (D) of HA-TRIM25.

**Figure 5:**
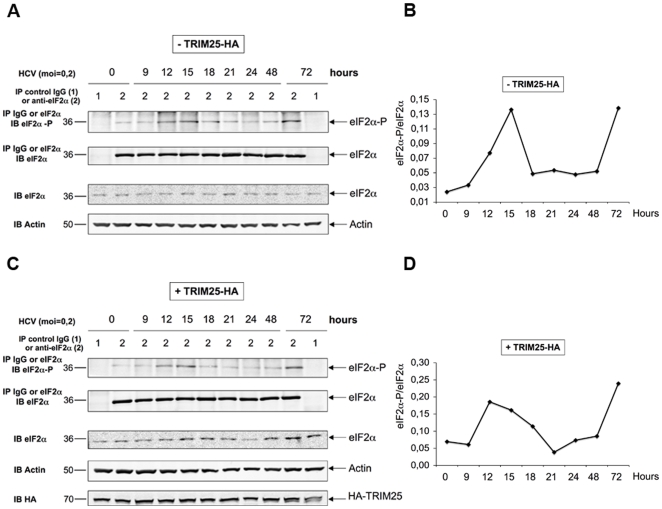
HCV activates the phosphorylation of eIF2α at 12 and 15 hrs post infection. The detection of eIF2α and eIF2α-P and the quantification of the ratio eIF2α-P/eIF2α in the absence (**A and B**) or in the presence (**C and D**) of HA-TRIM25 was performed as described in the legend to Figure 4.

The results shown in Figure 3 suggested a control of IFN expression by HCV at the protein level. In addition to this, an analysis of the effect of infection on the activity of a different luciferase reporter construct (TK-Renilla luciferase; Figure S1) revealed a similar specific inhibition at 12 hrs post-infection with HCV and not with Sendaï virus, indicating that HCV could exert a control on protein expression at the general translational level. One possible candidate that could exert such a control is the protein kinase PKR, which inhibits protein synthesis through phosphorylation of the initiation factor eIF2α [13]. Activation of the catalytic domain of PKR is mediated by a change in its conformation due to the interaction of its N terminus with dsRNA structures or with specific proteins [10]. The fact that IFN could be induced during the first 12 hrs post-infection with JFH1 in Huh7.25.CD81/TRIM25 or in Huh7.5/RIG-I cells was an indication that HCV dsRNA structures had appeared in the cytosol that activate RIG-I and that these structures may also represent good candidates to activate PKR. We therefore examined the state and dynamics of phosphorylation of PKR and of its substrate eIF2α upon HCV infection, using Huh7.25/CD81 cells, either as such or upon ectopic expression of TRIM25. At different times post-infection, cell extracts were immunoprecipitated with antibodies directed against PKR or eIF2α, after which the degree of PKR or eIF2α phosphorylation was examined using anti-phospho PKR (residue Thr451) or anti-phospho eIF2α (residue Ser51) antibodies and by performing quantification of the p-PKR/PKR or the p-eIF2α/eIF2α ratio. The results clearly showed peaks of PKR (Figure 4) and eIF2α (Figure 5) phosphorylation as early as 12 and 15 hrs post-infection, followed by a decrease of phosphorylation until 24 hrs and a second increase until 72 hrs post-infection, the end-point of the experiment. The presence of TRIM25 was not found to significantly affect PKR or eIF2α phosphorylation, except for a less abrupt decline between15 and 24 hrs. Altogether, these results show that PKR and its substrate eIF2α are activated in the early hours of HCV infection.

### HCV infection triggers inhibition of protein translation

**Figure 6:**
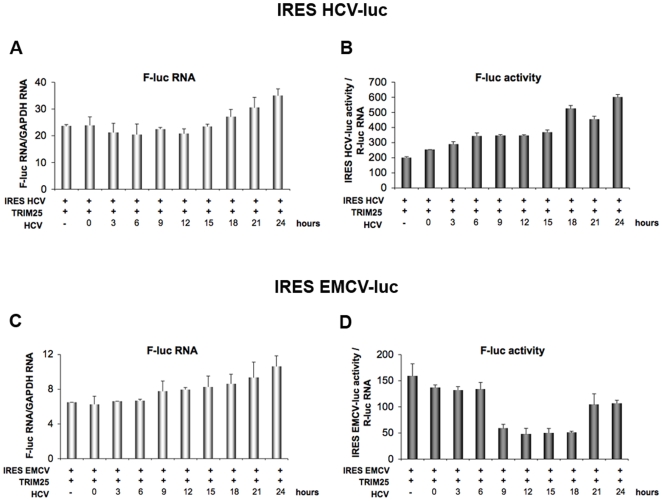
HCV triggers a transient inhibition of protein translation. Huh7.25.CD81 cells were transfected with 400 ng of CAT-IRES**^HCV^**-LUC (**A and B**) or 50 ng of CAT-IRES**^EMCV^**-LUC (**C and D**), together with the pRL-TK-RLUC plasmid (40 ng) and the HA-TRIM25 expressing plasmid (100 ng). 24 hrs post-transfection, cells were infected with JFH1 at an m.o.i of 0.2. **A and C**: At the indicated times, total cellular RNA was extracted and F-luc and GAPDH RNA were quantified by qRT-PCR. The graphs represent the number of copies of the IRES**^HCV^**-luc (A) or of the IRES**^EMCV^**-luc (C) RNA normalized to the number of copies of GAPDH RNA. Error bars represent the mean ± S.D. for triplicates. **B and D**: At the indicated times, cell lysates were prepared and the IRES**^HCV^**-luc or IRES**^EMCV^**-luc activity was analysed by a reporter assay. For normalization, the levels of firefly luciferase activity were divided in each case by the ratio R-luc RNA/GAPDH RNA that was calculated after measurement of the R-luc RNA by RTqPCR using the total cellular RNA extracted for A and C. Error bars represent the mean ± S.D. for triplicates.

HCV belongs to the family of viruses with 5′ IRES structures that are translated after direct binding of ribosomes to the RNA in the vicinity of the initiation codon. Interestingly, unlike most viral IRESs, translation from the HCV IRES was shown to be independent of eIF2α phosphorylation in situations of stress [14], [15]. To establish a correlation between the observed HCV-mediated PKR and eIF2α phosphorylation, and a possible inhibition of eIF2α-dependent translation by HCV, we next compared the effects of HCV infection on the kinetics of expression of a luciferase reporter placed under the control of the IRES**^HCV^** (insensitive to eIF2α phosphorylation) or under that of the IRES **^EMCV^** (sensitive to eIF2α phosphorylation). The experiment was done in Huh7.25/CD81 cells transfected with a TRIM25-expressing plasmid, to perform the analysis in conditions leading to IFN induction. For each IRES-luc expressing plasmid, we took care to choose concentrations of these plasmids that give similar levels of RNA expression or luciferase activity (data not shown and Figure 6). After transfection by the different plasmids, the cells were infected with JFH1 and the levels of RNA or luciferase expression followed over 24 hrs. The expression levels of IRES **^EMCV^** RNA and IRES **^HCV^** RNA were found to increase similarly with time post-infection (Figures 6A and C). In contrast, there was a dramatic difference in the expression levels of luciferase expressed from the IRES **^EMCV^** compared to that expressed from the IRES **^HCV^** (Figures 6B and D). Expression of luciferase from the IRES **^EMCV^** was strongly inhibited at 12 hrs post-infection with JFH1, after which time it was progressively restored until at 24 hrs post-infection it approached its initial level. In contrast, expression of luciferase from the HCV IRES was not inhibited at any time after infection with JFH1. Rather, it steadily increased with time post-infection. Similarly to the experiment presented in Figure 3, concomitant analysis of luciferase activity placed under a TK promoter revealed inhibition at 12 hrs post-infection with HCV, regardless of the presence of IRES **^EMCV^** or IRES **^HCV^**, again indicating that the inhibition effect is due to HCV infection and occurs at a general level (Figure S2). These results demonstrate that HCV infection triggers inhibition of general translation, while translation from the IRES **^HCV^** is unaffected. Since inhibition of translation occurs at 12 hrs post-infection, at a time when PKR and eIF2α are phosphorylated (Figures 4 and 5), this shows a correlation between an HCV-mediated inhibition of protein synthesis and PKR/eIF2α phosphorylation; a hallmark of eIF2α-dependent translation.

### Depletion of PKR increases IFN induction

**Figure 7:**
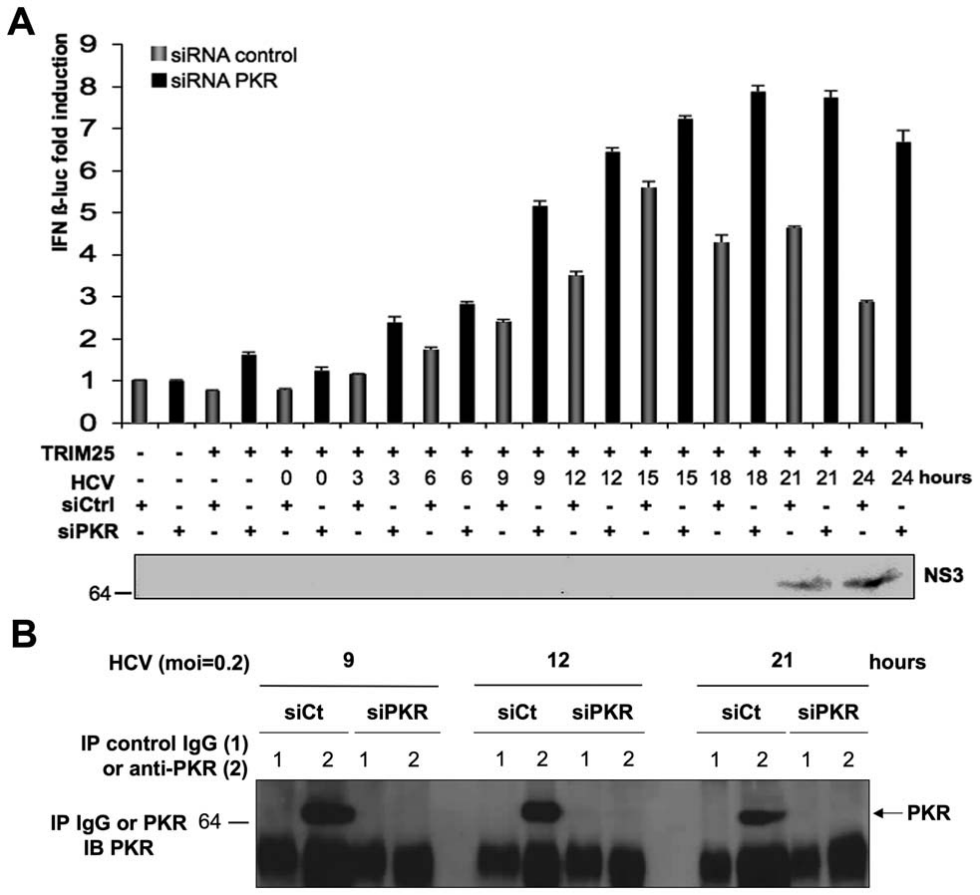
Depletion of PKR increases IFN induction in HCV-infected cells. Huh7.25.CD81 cells were first transfected with 25 nM of siRNA directed against PKR or with 25 nM of control siRNA and then transfected 24 hrs later with the pGL2-IFNβ-FLUC/pRL-TK-RLUC reporter plasmids and the TRIM25 expressing plasmid. 24 hrs post-transfection the cells were infected with JFH1 at an m.o.i. of 0.2. **A**: At the indicated times, one set of cells was treated for RNA extraction and the other for reporter assay as described in the legend to Figure 3. IFN expression was expressed as fold-induction over control cells that were simply transfected with pGL2-IFNβ-FLUC/pRL-TK-RLUC and either control siRNA or siPKR. The graph represents the levels of firefly luciferase activity normalized to the ratio R-luc RNA/GAPDH RNA. Error bars represent the mean ± S.D. for triplicates. In addition, cell lysates were pooled and analysed for the presence of NS3 as a marker of HCV infection. **B**: Huh7.25.CD81 cells were treated as in A, except for transfection with the reporter plasmids, to control the efficiency of PKR silencing. At the indicated times post-infection, cell extracts (1.7 mg) were incubated with normal mouse IgG or Mab 71/10 anti-PKR. The immunoprecipitated complexes were run on SDS-12.5% PAGE gels and blotted using Mab 71/10 (PKR).

We next examined further the role of the ability of HCV to activate PKR in the early events of infection, in relation to IFN induction. Huh7.25/CD81 cells were transfected with control siRNAs or siRNA directed against PKR, in conditions that allow complete depletion of PKR (Figure 7B). They were then transfected with TRIM25 in the presence of the IFNβ-luciferase reporter plasmid. Cells that express normal endogenous levels of PKR presented an initial increase in IFNβ-luciferase induction up to 12–15 hrs in response to infection with JFH1 followed by a sudden decline (Figure 7A), similarly to the results described in Figure 3. In contrast, induction of IFNβ-luciferase continued to increase past 15 hrs post-infection in cells in which PKR expression was silenced. In both cases, a decrease of IFN expression at 24 hrs was correlated with strong NS3 expression in the cytosol (Figure 7A). Concomitant analysis of the activity of luciferase placed under a TK promoter revealed the same pattern of inhibition of expression during HCV infection, which was alleviated when PKR was silenced. This confirmed that HCV-mediated inhibition at the general level occurs through PKR (Figure S3). Therefore, these results demonstrate the role of PKR activation in controlling IFN induction in HCV infection prior to MAVS cleavage by the HCV NS3/4A.

### PKR positively controls HCV yields through the inhibition of the IFN induction pathway

**Figure 8:**
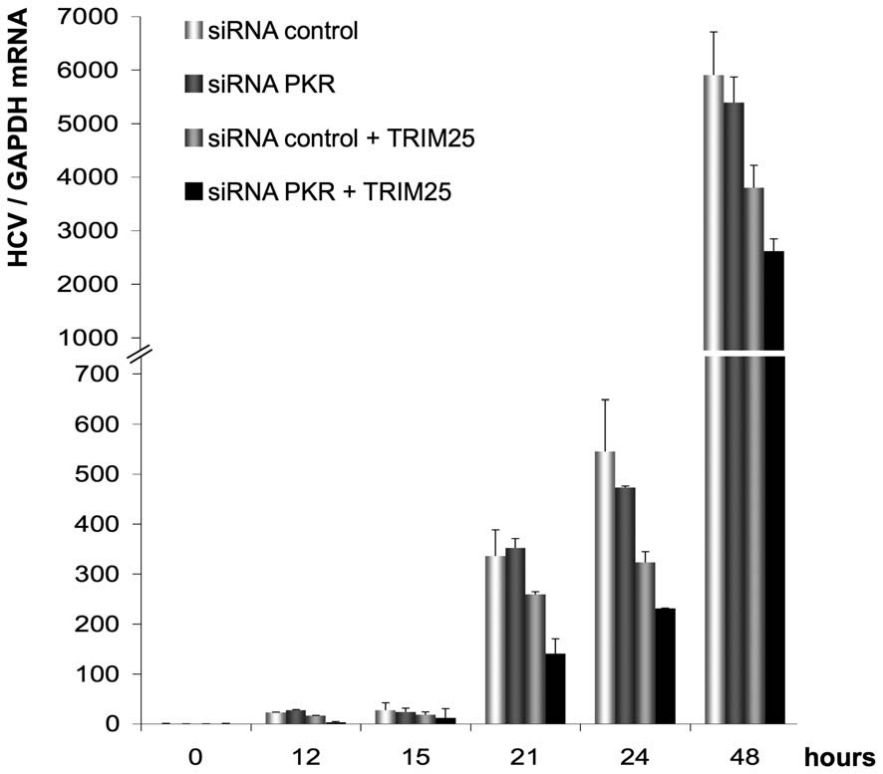
PKR positively controls HCV yield through inhibition of the IFN induction pathway. Huh7.25.CD81 cells were first transfected with 25 nM of siRNA directed against PKR or 25 nM of control siRNA and then transfected 24 hrs later with either empty vector or the TRIM25 expressing plasmid. 24 hrs post-transfection, the cells were infected with JFH1 at an m.o.i. of 0.2. At the indicated times, cells were processed for RNA extraction and HCV RNAs were quantified by qRT-PCR and normalized against RNA from GAPDH. Error bars represent the mean ± S.D. for triplicates.

We next examined whether there was a correlation between the control of IFN induction by PKR, and HCV yields. Huh7.25/CD81 cells were transfected with control siRNA or siRNA directed against PKR as in Figure 7, and infected with JFH1, in conditions where the IFN induction pathway was either active after transfection with TRIM25, or not (Figure 8). The virus yields were measured by RT-qPCR at different times post-infection. First, ectopic expression of TRIM25 triggered a significant inhibition of virus yields at 24 hrs post-infection (40%; Figure 8), as expected from its ability to restore IFN induction in Huh7.25/CD81 cells as shown in previous figures. Inhibition of virus yields did not increase later in infection (36% at 48 hrs post-infection; Figure 8), in agreement with the block on IFN induction downstream of TRIM25 imposed by the NS3/4A-mediated cleavage of MAVS. Depletion of PKR from Huh7.25/CD81 cells also triggered inhibition of virus yields. Interestingly, whereas this inhibition was limited in the absence of TRIM25 (13% and 9% inhibition at 24 and 48 hrs post-infection, respectively), it was more pronounced when the cells were expressing TRIM25, with a 28% and 31% further increase in inhibition after 24 and 48 hrs of infection, respectively (Figure 8). Altogether, the combined effect of TRIM25 expression and PKR depletion resulted in 58% of inhibition of HCV virus yields at 24 hrs and 56% at 48 hrs post-infection. These data demonstrate that HCV infection is particularly sensitive to the IFN induction pathway, and that the efficiency of infection benefits from an inhibition of this pathway at the translational level by PKR.

### Pharmacological inhibitors of PKR increase IFN induction and inhibit HCV infection

**Figure 9:**
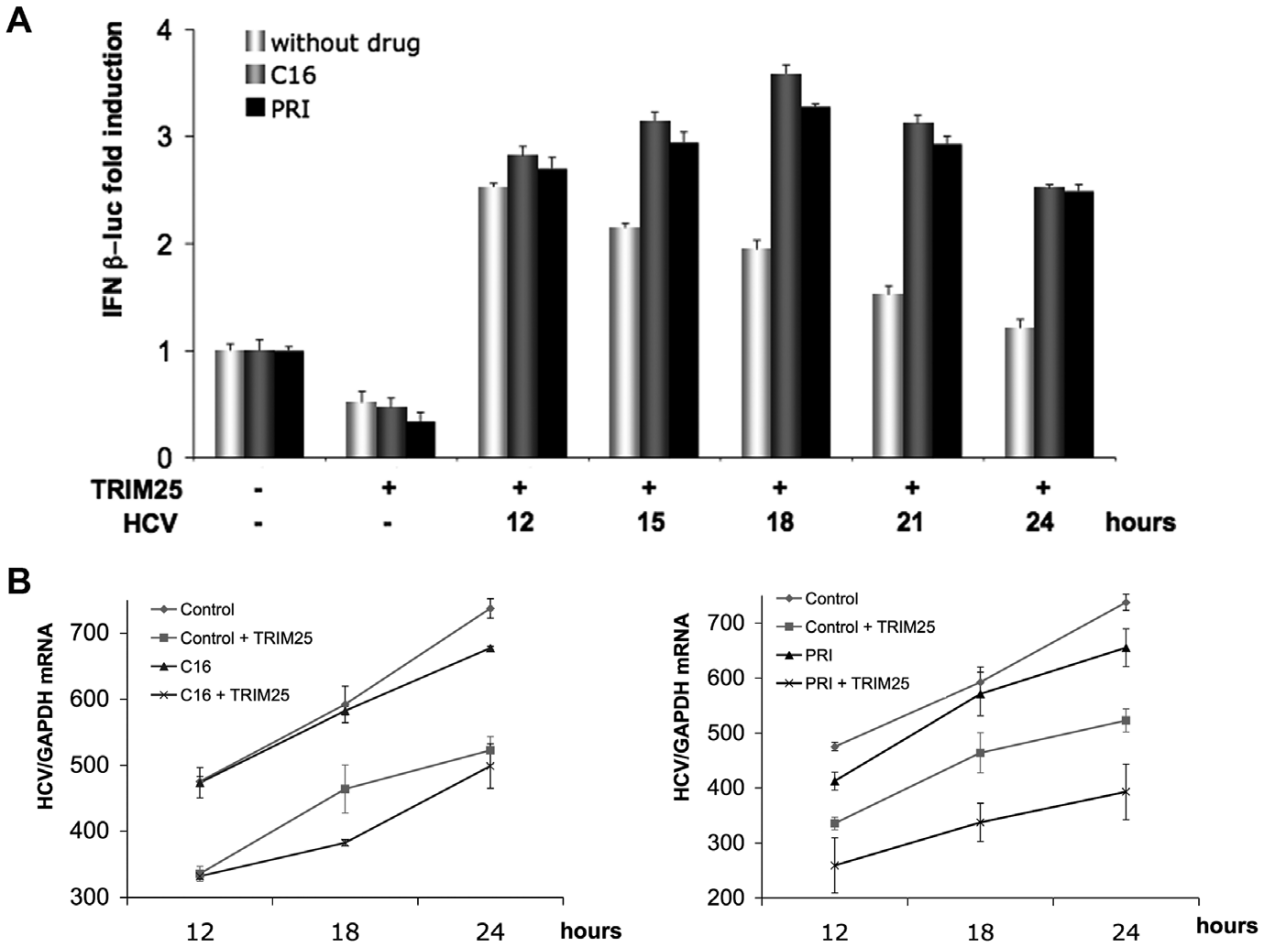
Pharmacological inhibitors of PKR increase IFN induction and inhibit HCV infection. **A**: Huh7.25.CD81 cells were first transfected with the pGL2-IFNβ-FLUC/pRL-TK-RLUC reporter plasmids and the TRIM25 expressing plasmid. 24 hrs post-transfection, the cells were infected with JFH1 at an m.o.i of 0.2. At 11 hrs post-infection, cells were exposed to 200 µM of C16 or 30 µM of the PRI peptide as described in Materials and Methods. At the indicated times, one set of cells was treated for RNA extraction and the other for reporter assay as described in the legend to Figure 3. IFN expression was expressed as fold-induction over control cells that were simply transfected with pGL2-IFNβ-FLUC/pRL-TK-RLUC. The graph represents the level of firefly luciferase activity normalized to the ratio R-luc RNA/GAPDH RNA. Error bars represent the mean +/- S.D. for triplicates. **B**: Huh7.25.CD81 cells were first transfected with the TRIM25 expressing plasmid or an empty plasmid. 24 hrs post-transfection, the cells were infected with JFH1 at an m.o.i of 0.2. At 11 hrs post-infection, cells were exposed to 200 µM of C16 or 30 µM of the PRI peptide as described in Materials and Methods. At the indicated times, cell lysates were processed for RNA extraction and HCV RNAs were quantified by qRT-PCR and normalized against RNA from GAPDH. Error bars represent the mean +/- S.D. for duplicates.

Although best known for its antiviral proprieties, PKR is now also recognized for its negative effect on neurodegenerative disorders such as Huntington's, Parkinson's and Alzheimer's diseases [16]. For these reasons, pharmacological inhibitors of PKR have been developed. Here, we have used two PKR inhibitors, the oxindole-imidazole C16 compound [17] and the cell penetrating PRI peptide, that corresponds to one PKR motif (DRBM) involved in binding to its activators. This latter compound represents a more specific PKR inhibitor than C16 [18]. We examined the effects of these inhibitors on IFN induction and HCV replication during infection of Huh7.25/CD81 cells with JFH1 (Figure 9). The cells were transfected with TRIM25 as described in Figure 7, and incubated with either C16 or PRI after 11 hrs of infection. The results clearly show that both PKR inhibitors increased IFNβ promoter induction (Figure 9A), as well as restoring general level of protein expression (Figure S4). This increase was more pronounced at 15–18 hrs post-infection (Figure 9A). This highlights the importance of PKR activation during this time-frame to control IFN induction. Next, the effect of the PKR inhibitors on HCV replication was assayed. Huh7.25/CD81 cells were transfected with TRIM25, or not, to have a non-functional or functional RIG-I/MAVS pathway. Similarly to the experiment shown in Figure 8, PKR inhibitors were able to inhibit HCV yields only in conditions where the RIG-I/MAVS pathway was activated (Figure 9B).

## Discussion

Here we report the use of HCV permissive Huh7.25.CD81 cells to analyse the effect of HCV on the RIG-I/MAVS-mediated IFN induction pathway during the early hours after infection, before the HCV NS3/4A protease can cleave MAVS and abrogate this pathway. We first observed that the IFN induction pathway was deficient in these cells, but that it could be conveniently restored upon ectopic expression of the E3 ubiquitin ligase TRIM25. This protein has been shown to bind the threonine 55 residue of RIG-I through its C terminal SPRY domain, and uses the E3 ligase activity present in its N terminal RING domain to promote a Lys63-type ubiquitination of RIG-I in its CARD domain at lysine 172. This modification is important if RIG-I is to engage in a correct interaction with its downstream adapter MAVS [3]. The nature of the TRIM25 defect in Huh7.25.CD81 cells and the relation to activation of RIG-I is now under characterization. It is interesting to note here that this defect emphasizes the importance of the RIG-I/TRIM25 pathway in the control of HCV expression since both Huh7.25.CD81 and Huh7.5 cells, which were originally selected for their ability to replicate HCV subgenomic replicons, present a defect in this pathway at the level of RIG-I for Huh7.5 cells and of TRIM25 for Huh7.25.CD81 cells. Importantly, Huh7.25.CD81 cells can be considered as a more physiological model than Huh7.5 cells to study the interaction between the IFN induction pathway and HCV infection, since Huh7.5 cells have a point mutation at the threonine 55 residue of RIG-I [5] and therefore require ectopic expression of RIG-I to restore their IFN induction pathway. In contrast, ectopic expression of TRIM25 restores the IFN induction pathway downstream of RIG-I and therefore does not perturb the initial interaction between RIG-I and the incoming HCV dsRNA structures.

Using Huh7.25.CD81 cells that ectopically express TRIM25, we observed that HCV triggers IFNβ induction as early as 6 hrs after infection, demonstrating that the entry of the virus into the cell rapidly generates enough viral structured RNAs to activate RIG-I. NS3/4A expression was detected around 18 hrs post-infection, and MAVS cleavage after 24 hrs post-infection. We thus expected to observe an increase in IFNβ induction at least until 18 hrs after infection. This was indeed found to be the case when we analysed IFNβ induction at the RNA level. However, when we used a luciferase reporter gene placed under the IFNβ promoter as a surrogate marker for production of the IFNβ protein, it was intriguing to observe an arrest of IFN induction as rapidly as 12 hrs after infection, followed by a considerable decline in its expression afterwards. These results indicated a new control of IFN induction at the translational level during HCV infection in addition to –and earlier than- the NS3/4A-mediated MAVS cleavage. We have demonstrated that this inhibition is the result of a strong activation of the protein kinase dsRNA-dependent kinase PKR at 12 and 15 hrs after HCV infection and of the concomitant phosphorylation of its substrate eIF2α.

Since this manuscript was submitted, work performed by U.Garaigorta & F. Chisari showed that HCV also activates PKR and eIF2α phosphorylation to negatively control the antiviral action of IFN[19]. Both their and our data concur to point out that HCV diverts PKR away from its known antiviral role to use it to attenuate both the IFN-mediated antiviral response [19] and IFN induction itself (our data). Importantly, both studies show that PKR silencing has no effect on HCV production, unless cells are allowed to induce IFN upon ectopic expression of TRIM25 (our study) or are directly treated with IFN [19].

PKR is a 551 aa serine/threonine protein kinase with its catalytic domain at the C-terminus and regulating domains at the N-terminus. This N-terminus domain contains two tandem copies of a 70-amino acid dsRNA binding motif or DRBM (positions 6–79 and 96–169) that affiliate PKR to the large family of dsRNA binding proteins, which includes TRBP, PACT, DICER, ADAR, RNAse III and NFAR's [20]. Binding of PKR to dsRNA induces its dimerization and a change of its conformation that frees access to its autophosphorylation site. Structural analysis has revealed that PKR dimers are arranged back to back, which prevents trans-phosphorylation of one monomer by the other and favours a model of autophosphorylation in cis or through the action of a PKR dimer on other PKR dimers or monomers [21]. Once autophosphorylated on its threonine T446, PKR is stabilized as a kinase and can phosphorylate its substrates, such as the α subunit of the initiation factor eIF2. PKR is preferentially activated upon binding to long stretches of dsRNA (>33 bp) [22] but was recently shown also to be activated upon binding to RNA structures similar to those that activate RIG-I, such as short imperfect 16 bp stem-loop RNAs with 5′ triphosphorylated ends. It is now proposed that perfect dsRNAs can activate PKR with no need for a 5′ppp, whereas if they are to activate, dsRNA with minimal secondary structures are dependent on 5′ppp [23]. In addition, PKR activity can be regulated positively and negatively through interaction with the cellular proteins PACT and TRBP, two members of the dsRNA binding protein family. This type of interaction, which is independent of the presence of dsRNA, is complex. The TRBP/PACT interaction can modulate the activation of PKR by PACT and a cellular stress reverses it [24].

Our results point out the ability of HCV to trigger both induction of IFN and activation of PKR in the early hours of infection. The induction of IFN is initiated by the binding of RIG-I to viral 5′ppp structured RNA, the 3′ end poly-U/UC motif and/or replicative RNA duplexes [5], [25]. In vitro experiments have shown that PKR can be activated upon binding to the HCV IRES [26] but it is currently not known exactly how HCV activates PKR in vivo. The use of antibodies directed against phosphorylated PKR or against phosphorylated eIF2α allowed in vivo activation of PKR to be demonstrated, either upon HCV subgenomic replicon replication [27] or more recently after electroporation of Huh7 cells with JFH1 RNA [28]. Surprisingly, the latter study showed that silencing of PKR resulted in 10-fold higher viral yields compared to our study (Figures 8 and 9) and that of Garaigorta & Chisari [19], where no effect on viral yields could be observed unless the IFN induction pathway is triggered or IFN is added to the cells. Although it is difficult to compare different experimental systems, the discrepancy observed may be due to the different procedures used to express JFH1 in the cells: electroporation of its RNA in Kang'study, compared to infection with JFH1 viral stocks in our study and that of Garaigorta & Chisari. Interestingly, the differences might indicate that the mode and/or efficiency of interaction between the HCV structures required to activate PKR are crucial to determine how the virus will benefit or suffer from PKR action. PKR has also been shown to be activated through an interaction between its N terminal domain and the first 58 amino acids of HCV core protein [29]. Core is the first HCV protein to be processed upon translation of the viral polyprotein and may interact with endogenous PKR soon after its own apparition in the cytosol. The possibility evoked here that core is involved in PKR activation requires further investigation.

Our results show that the IFN induction pathway can be stimulated during HCV infection up to 12 hrs post-infection, followed by a decline concomitantly with activation of PKR and of its substrate eIF2α. Importantly, depletion of the endogenous PKR by silencing, or inhibition of its function through the use of pharmacological inhibitors, prevents this decline. This indicates that HCV infection triggers an inhibition of general protein synthesis through PKR, whereas its own translation is not affected, as shown by the appearance of the viral proteins and an increase in expression of a reporter gene placed under the control of an IRES **^HCV^**. These data are in agreement with a recent study which showed, using in vitro experiments, that PKR can inhibit both cap-mediated and IRES **^EMCV^**-mediated translation, but not IRES **^HCV^**-mediated translation [26]. Shimoike et al proposed that HCV uses alternate initiation factors that allow a mechanism of translation initiation distinct from other IRES and from the cellular machinery of general translation that is not inhibited by eIF2α phosphorylation. The resistance of IRES **^HCV^** to control of translation may be related to a positive effect *in trans* from the virus through some of its structures, such as the polyadenylated 3′ UTR [30]. Alternatively, translation from the HCV IRES may occur in autophagy-like structures, together with translation from the incoming HCV RNA, where it may escape PKR action [31]. By both escaping and down regulating host cap-dependent translation, HCV may gain a further selective advantage for its own replication and propagation.

**Figure 10:**
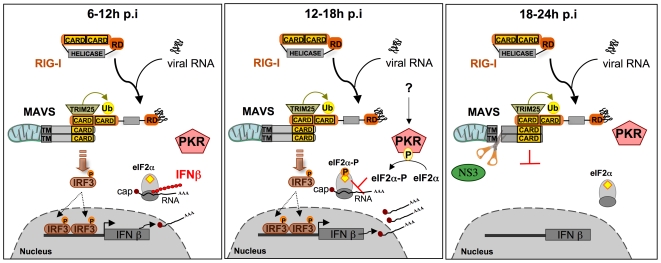
Model of regulation of IFN β induction at the very early steps of HCV infection. **Left panel**: *6–12 hrs after infection*. After cell entry, the HCV genomic RNA is liberated, and thus dsRNA structures are accessible in the cytosol and can associate with the RIG-I RNA helicase. This triggers activation of RIG-I followed by its ubiquitination by the E3-ligase TRIM25, and interaction with the mitochondria-bound MAVS adapter. MAVS, in turn, activates downstream signalling kinases leading to IRF3 phosphorylation and induction of IFNβ. Detection of IFNβ expression starts at 6 hrs p.i. and increases until 12 hrs post-infection. Induced IFNβ mRNA moves to the cytosol and is translated into IFNβ protein through the cap-dependent process of cellular translation. **Middle panel**: *12–18 hrs after infection*. HCV infection triggers the activation of PKR through a still unknown mechanism (either through its dsRNA or through a viral protein, such as core, or through activation of a cellular protein, such as PACT). Once activated, PKR phosphorylates its substrate, the α subunit of the eIF2 initiation complex and arrests the cap-dependent protein synthesis. As a result, translation of IFNβ protein stops. **Right panel**: *18–24 hrs after infection.* HCV proteins have been translated through a cap-independent mechanism and begin to accumulate in the cytosol. The viral NS3 protease cleaves MAVS at residue 508. This results in complete inactivation of MAVS, arrest in the recruitment of its downstream kinases and arrest of IFN induction. Note that after 15 hrs, PKR is no longer phosphorylated, hence no longer activated. The mechanism of this regulation is not yet known. RIG-I: Retinoic acid Inducible Gene 1; CARD: Caspase Recognition Domain; RD: Repressor Domain, Ub:Ubiquitin; MAVS: Mitochondria Adaptor Virus Signalling; TM: Transmembrane domain; PKR: Protein Kinase RNA-dependent; IRF3: Interferon Regulatory Factor 3; eIF2α: α subunit of eukaryotic Initiation Factor; AAA: polyadenylated tail. P: phosphate group.

Interestingly, we found that the depletion of PKR from Huh7.25.CD81 cells, or incubation with the PKR inhibitors C16 and PRI, leads to inhibition of HCV replication, but only under conditions where the IFN induction pathway has been restored by the expression of TRIM25. Our results thus demonstrate that, among all the cap-dependent proteins the expression of which are sensitive to PKR, only the translation of proteins resulting from the activation of the IFN induction pathway represent a real constraint for HCV replication and propagation. Early activation of PKR upon HCV infection (12–15 hrs post-infection) allows this virus to limit the expression of IFN and pro-inflammatory cytokines as soon as possible, before enough NS3/4A is translated from the HCV IRES to block this signaling pathway through MAVS cleavage (18 to 24 hrs post-infection) (see model in Figure 10). Through detailed kinetic experiments, we demonstrated that phosphorylation of PKR and of its substrate eIF2α are restricted to a 3 hour-period between 12 and 15 hrs post-infection. Accordingly, the PKR inhibitors C16 and PRI efficiently restored IFN induction during this time. The use of both PKR silencing and the addition of pharmacological inhibitors of PKR demonstrates the importance of PKR in the control of IFN induction early after infection with HCV. These results highlight that the 3-hour period between 12 and 15 hrs post-infection is crucial for HCV to activate PKR to restrain IFN expression, once it has launched the IFN induction pathway through interaction of its replicative dsRNA forms with RIG-I. Its ability to activate PKR at the same time allows this induction to be moderated at the level of the translation of this cytokine before sufficient NS3/4A accumulates and abrogates IFN induction at the transcriptional level through MAVS cleavage.

It was intriguing to observe that PKR and eIF2α phosphorylation decrease at 15 hrs post-infection with HCV, to reappear progressively and increase from 24 hrs post-infection until the 72 hr end-point of the experiment (Figures 4 and 5). The reason for this remains to be investigated and may indicate that PKR could be sensitive to the action of phosphatase(s) at certain time points of the infection.

PKR is well-recognized as an important effector of the antiviral response through its ability to arrest protein synthesis and its importance is highlighted by the number of viral and cellular products that are able to abrogate or modulate its action [13], [32]. In the case of HCV, some viral proteins such as NS5A and a cytosolic soluble form of E2 were reported to interact with PKR, and were proposed to be viral inhibitors of the antiviral action of PKR [33], [34], [35].

Here, we show another aspect of the interaction of HCV with PKR, that reveals how HCV uses the ability of PKR to inhibit cellular cap-dependent translation, while avoiding its effect through its own translation driven by the HCV IRES. PKR silencing or PKR inhibitors have less effect on HCV production when the RIG-I/MAVS pathway is non-operative. This demonstrates that it is only IFN (and presumably also the pro-inflammatory cytokines resulting from the activation of the RIG-I/MAVS pathway) that represents a threat for viral propagation. The situation we describe here might correspond to primo-infection during the natural course of HCV infection in an IFN-free environment, allowing unabated propagation of the virus due to very limited production of IFN. In established chronic infections, whatever IFN has been produced can then trigger induction of a large number of ISGs (IFN-stimulated genes), such as RIG-I and TRIM25, which can sustain the IFN induction pathway, and ISG56 or viperin, which are involved in the inhibition of HCV translation and replication [36], [37]. Although PKR is also recognized in several viral infections for its antiviral proprieties, it may be more suitable to control its action in the case of HCV infection. Therefore, we propose that a carefully-controlled use of PKR inhibitors, in conjunction with IFN/ribavirin, would be beneficial for the treatment of chronically HCV-infected patients, since it would lead to a boost in the induction of innate immunity and a sustained inhibition of the viral propagation.

## Materials and Methods

### Cell culture

Huh7 and Huh7.5 cells were cultured in Dulbecco's modified Eagle's Medium (DMEM; Sigma) supplemented with 10% heat-inactivated fetal bovine serum, 1% nonessential amino acids (Gibco BRL) and 1% penicillin/streptomycin (Invitrogen). Huh7.25/CD81 cells were cultured in the same medium supplemented with 0.4 mg/ml geneticin (G418; PAA Laboratories).

### Viruses

Sendai virus stocks were grown in the allantoic cavities of 9-day-old embryonated chicken eggs that were provided by D. Garcin, as described [38]. For the preparation of HCV stocks, Huh7.5 cells plated in 150 cm^2^ flasks were inoculated with JFH1 at an m.o.i of 0.05, in serum-free DMEM. After 2 hrs at 37°C, the medium was removed and replaced with complete medium. Three days after infection, the cells were dissociated by trypsin treatment, and replated in 3 or 4 150 cm^2^ flasks. After 3 more days, the JFH1-containing cell supernatants were collected, centrifuged at 1200 rpm for 10 min at 4°C, cleared of debris by passing through 0.45-µm-pore-size filters, and stored at −80°C. The cells were trypsinated once again, passaged in 1 or 2 150 cm^2^ flasks and viral supernatants were collected again after 24 hrs and stored at −80°C. For titration, Huh7.5 cells (10^5^ cells/well in a 24-well plate) were incubated in the presence of serial dilutions of the viral preparations. After three days, cells were washed with PBS, fixed for 20 min with 4% paraformaldehyde-PBS (PFA; Electron Microscopy Sciences), permeabilized by incubation for 1 hour with PBS that contained 1% gelatin (Sigma) and 0.1% Tween-20 (Sigma), and incubated for 1 hour with 150 µl of PBS-gelatin that contained serum from a patient chronically infected with HCV (genotype 1b) at a 1/500 dilution. Cells were washed and incubated with peroxidase-conjugated anti-human antibody (1/2500 dilution; Dako), and the presence of the virus was detected using the Vector NovaRED substrate kit for peroxydase (VECTOR). Infectious foci were counted under a microscope and the titer was calculated as focus forming units per ml. Titers of 6.10^4^ FFU/mL were obtained on a routine basis.

### Plasmids, siRNAs and antibodies

The pcDNA3 expressing 5′HA tagged-TRIM25 was provided by D. Garcin. The CAT-IRES**^HCV^**-LUC and CAT-IRES**^EMCV^**-LUC constructs have been described [39]. The IFNβ-firefly luciferase (pGL2-IFNβ) and pEF-BOS FLAG-RIG-I plasmids were described previously [40]. The pcDNA3.1 cMyc-MAVS has been described [41]. Control (scrambled) siRNA and siRNA to PKR (GCAGGGAGUAGUACUUAAAUAUU) were synthesized by Dharmacon Research, Inc. (Lafayette, CO). The siRNAs (final concentration 25 nM) were transfected for 24 h using FuGENE HD (ROCHE) before the transfection with other plasmids, or infection. Normal mouse IgG were from Santa Cruz Biotechnology. Monoclonal anti-PKR 71/10 antibody was obtained from A.Hovanessian [42]. Anti-actin antibody was from Sigma. Rabbit polyclonal antibodies were used to detect eIF-2α phosphorylated at Serine 51 (Cell Signalling Technology), PKR phosphorylated at Thr451 (AbCAM), and MAVS (Alexis Biochemical Inc.). Mouse monoclonal antibodies were used to detect HCV NS3 and the HA tag (Chemicon and 12CA5; Roche, respectively).

### Luciferase Assays

Huh7, Huh7.5 and Huh7.25.CD81 cells (80,000 cells/well; 24-well plates) were transfected with 40 ng of pRL-TK Renilla-luciferase (R-luc) reporter (Promega); 150 ng of pGL2-IFNβ-Firefly luciferase (F-luc) reporter, 400 ng of CAT-IRES**^HCV^**-Luc or 50 ng of CAT-IRES**^EMCV^**-Luc reporter; and pEF-BOS FLAG-RIG-I, pcDNA3 HA-TRIM25 or pcDNA3 cMyc-MAVS as indicated. Transfections were performed in the presence of the FuGENE HD reagent (ROCHE) according to the manufacturer's instructions. Cells were then incubated for 24 hrs before being mock-treated or infected with either SeV or JFH1. At different times post-infection, cells were processed for a reporter assay. For most of our experiments concerning activation of PKR and inhibition of general translation, normalization of the data was performed using Renilla luciferase RNA rather than its activity. All assays were performed in triplicate in two different sets of cells, plated, transfected and infected in the same conditions. For the reporter assay, the medium was removed and 100 µl of lysis buffer (Passive lysis buffer; Promega) was added to each well, and incubation was at room temperature for 15 minutes with gentle agitation. Luciferase activity was measured using the dual-luciferase reporter assay system, according to the manufacturer's instructions (Promega). RTqPCR analysis was performed on Renilla luciferase RNA and GAPDH RNA after extraction of total cellular RNA. The measured amount of Renilla luciferase RNA was normalized to the amount of GAPDH RNA and this ratio was used to normalize the Firefly luciferase activity.

To estimate the effficacy of transfection, Huh7.25.CD81 cells (80,000 cells/well; 24-well plates) were separately transfected with 1 µg of pcDNA3 Lac-Z in presence of FuGENE HD reagent. 24 hrs post-transfection, cells were fixed in 4% PFA and incubated for a few minutes with 200 µl of X-Gal stain (PBS, pH 7.2; 2 mM MgCl2; 4 mM potassium ferricyanide; 4 mM potassium ferrocyanide and 1 mg of X-Gal (Sigma) previously dissolved in DMSO). X-Gal-stained cells were counted in different sections of the plate and the cell density was determined as a function of the measured area of the section examined. Quantification from three fields taken at random allowed an estimation of 45% of the efficacy of transfection.

### Real-time RT-PCR Analysis

Total cellular RNA was extracted using the TRIZOL reagent, according to the manufacturer's instructions (Invitrogen). HCV RNA was quantified by one-step qRT-PCR. Reverse-transcription, amplification and real-time detection of PCR products were performed with 5 µl total RNA samples, using the SuperScript III Platinum one-step qRT-PCR kit (Invitrogen) and an AbiPrism 7700 machine. The sequence of the primers used for HCV RNA amplification are: 5′-TGCGGAACCGGTGAGTACA-3′ and 5′-CGGGTTGATCCAAGAAAGGA-3′, together with the internal probe 5′FAM- CGGAATTGCCAGGACGACCGG-3′TAMRA. IFN-β RNA, Renilla luciferase RNA and Firefly luciferase RNA were quantified by a two-step qRT-PCR assay. The reverse transcription step was performed on 1 µg of total RNA. Quantitative PCR was performed using an AbiPrism 7700 machine, with a SYBR GREEN PCR Master Mix (Applied BioSystemes). IFN-β was amplified using the primers: 5′-TGCATTACCTGAAGGCCAAG-3′ and 5′-AAGCAATTGTCCAGTCCCA-3′; Renilla luciferase was amplified using the primers: 5′-ATCATCCCTGATCTGATCGG-3′ and 5′-GGAGTAGTGAAAGGCCAGAC-3′ and Firefly luciferase with the primers: 5′-GAAGAGATACGCCCTGGTTCCT-3′ and 5′-TGTCCACCTCGATATGTGCATC-3′. Standard curves were established as described [40] using 10-fold serial dilutions of plasmids containing IFN-β, R-luc or F-luc amplicons. The results were normalized to the amount of GAPDH RNA using the GAPDH control kit from EuroGentec.

### Immunoprecipitation and Immunoblot analysis

Cells were washed once with phosphate-buffered saline and scraped into lysis buffer (50 mM TRIS-HCl, pH 7.5, 140 mM NaCl, 5% glycerol, 1% CHAPS) that contained cocktails of phosphatase and protease inhibitors (Complete, Roche Applied Science). The protein concentration was determined by the Bradford method. For immunoprecipitation, lysates were incubated at 4°C overnight with monoclonal anti-PKR 71–10 antibody or anti-eIF2α antibody, and then in the presence of A/G-agarose beads (Santa Cruz Biotechnology) for 60 minutes. The beads were washed three times, and the precipitated proteins were extracted at 95°C using SDS-PAGE sample buffer. Protein electrophoresis was performed on standard SDS-PAGE or NuPAGE gels (Invitrogen). Proteins were respectively transferred onto polyvinylidene difluoride membranes (BioRad) or nitrocellulose membranes (Biorad), and probed with specific antibodies. Immunoblots were revealed using either the ECL Plus Western Blotting Detection System (Amersham Biosciences) or chemiluminescence. In the latter case, nitrocellulose membranes were blocked in Odyssey Blocking Buffer (Rockland) for one hour and incubated with primary antibodies in Odyssey Blocking Buffer with gentle shaking. They were then incubated for 35 mn in the presence of appropriate secondary antibodies labelled with IRDye 800 (Rockland Immunochemicals), with gentle shaking and protection from light. Fluorescent immunoblot images were acquired and quantified by using an Odyssey scanner and the Odyssey 1.2 software (Li-Cor Biosciences).

### Chemical treatments

The cell-permeable peptide containing the DRBM of double-stranded RNA-dependent protein kinase (PRI peptide [18]; Sanofi-Aventis, Vitry sur Seine, France; dissolved in water), and the C16 compound ([17]; Calbiochem; solubilized in DMSO), were provided by Jacques Hugon. Huh7.25.CD81 cells were infected by JFH1 for 11 hrs, washed once with phosphate buffered saline and then exposed to serum-free DMEM containing either 200 µM of C16 or 30 µM of PRI peptide. In the latter case, the cells received fresh aliquotes of the PRI peptide (30 µM final concentration) every hour until the end of treatment to maximise the effect of the peptide. At different times after infection, cells were lysed and analysed by reporter assay or real-time RT-PCR as decribed above.

## Supporting Information

Figure S1 Specific inhibition of TK-Renilla luciferase activity 12 hrs post-infection with HCV. The graphs represent the R-luc activity normalized to R-luc RNA in the cell extracts corresponding to the experiment described in Figure 3, in which Huh7.25.CD81 cells (A) or Huh7.5 cells (B) have been infected with either SeV (top) or HCV (bottom). Error bars represent the mean ± S.D. for triplicates.
(1.19 MB TIF)

Figure S2 Inhibition of TK-Renilla luciferase activity 12 hrs post-infection with HCV in the presence of an IRES either from HCV or from EMCV. The graphs represent R-luc activity normalized to R-luc RNA in the cell extracts corresponding to the experiment described in Figure 6, in which Huh7.25.CD81 cells have been transfected with 400 ng of CAT-IRESHCV-LUC (A) or 50 ng of CAT-IRESEMCV-LUC (B), together with the pRL-TK-RLUC plasmid (40 ng) and the HA-TRIM25 expressing plasmid (100 ng). Error bars represent the mean ± S.D. for triplicates.
(0.15 MB TIF)

Figure S3 Silencing of endogenous PKR abrogates HCV-mediated inhibition of TK-Renilla luciferase activity. The graphs represent R-luc activity normalized to R-luc RNA in cell extracts corresponding to the experiment described in Figure 7, where Huh7.25.CD81 cells were first transfected with 25 nM of siRNA directed against PKR or with 25 nM of control siRNA and then transfected 24 hrs later with the pGL2-IFNβ-FLUC/pRL-TK-RLUC reporter plasmids and the TRIM25 expressing plasmid. 24 hrs post-transfection, the cells were infected with JFH1 at an m.o.i. of 0.2. Error bars represent the mean ± S.D. for triplicates.
(0.71 MB TIF)

Figure S4 Pharmacological inhibitors of PKR abrogate the HCV-mediated inhibition of TK-Renilla luciferase activity. The graphs represent R-luc activity normalized to R-luc RNA in cell extracts corresponding to the experiment described in Figure 9A, in which Huh7.25.CD81 cells were first transfected with the pGL2-IFNβ-FLUC/pRL-TK-RLUC reporter plasmids and the TRIM25 expressing plasmid. 24 hrs post-transfection, the cells were infected with JFH1 at an m.o.i of 0.2. At 11 hrs post-infection, cells were exposed to 200 µM of C16 or 30 µM of the PRI peptide. Error bars represent the mean ± S.D. for triplicates.
(0.99 MB TIF)
