# Supplementary figures and images for: Hepatitis C Virus Controls Interferon Production through PKR Activation
Source: PLoS One. 2010 May 11;5(5):e10575. doi: 10.1371/journal.pone.0010575 (PMC2868028; doi:10.1371/journal.pone.0010575)

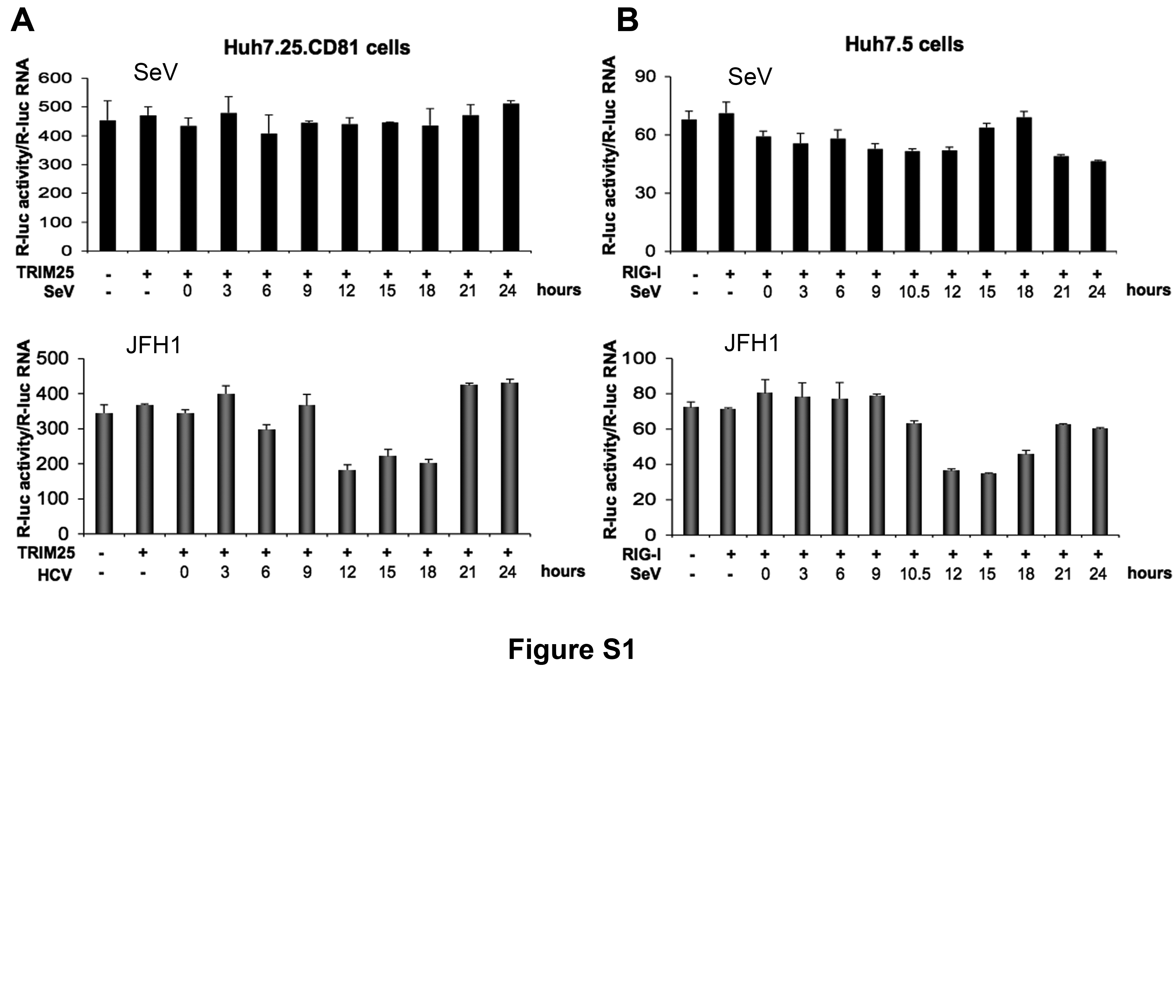

Supplement: Figure S1 — Specific inhibition of TK-Renilla luciferase activity 12 hrs post-infection with HCV. The graphs represent the R-luc activity normalized to R-luc RNA in the cell extracts corresponding to the experiment described in Figure 3, in which Huh7.25.CD81 cells (A) or Huh7.5 cells (B) have been infected with either SeV (top) or HCV (bottom). Error bars represent the mean ± S.D. for triplicates. (1.19 MB TIF) [file pone.0010575.s001.tif]

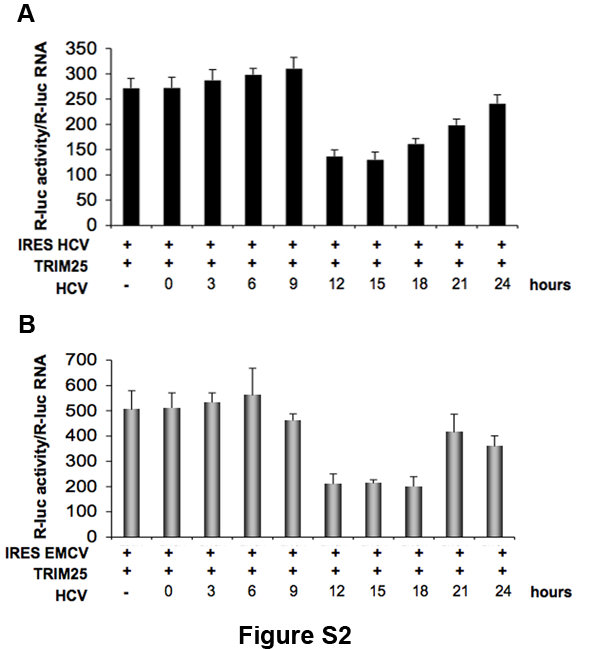

Supplement: Figure S2 — Inhibition of TK-Renilla luciferase activity 12 hrs post-infection with HCV in the presence of an IRES either from HCV or from EMCV. The graphs represent R-luc activity normalized to R-luc RNA in the cell extracts corresponding to the experiment described in Figure 6, in which Huh7.25.CD81 cells have been transfected with 400 ng of CAT-IRESHCV-LUC (A) or 50 ng of CAT-IRESEMCV-LUC (B), together with the pRL-TK-RLUC plasmid (40 ng) and the HA-TRIM25 expressing plasmid (100 ng). Error bars represent the mean ± S.D. for triplicates. (0.15 MB TIF) [file pone.0010575.s002.tif]

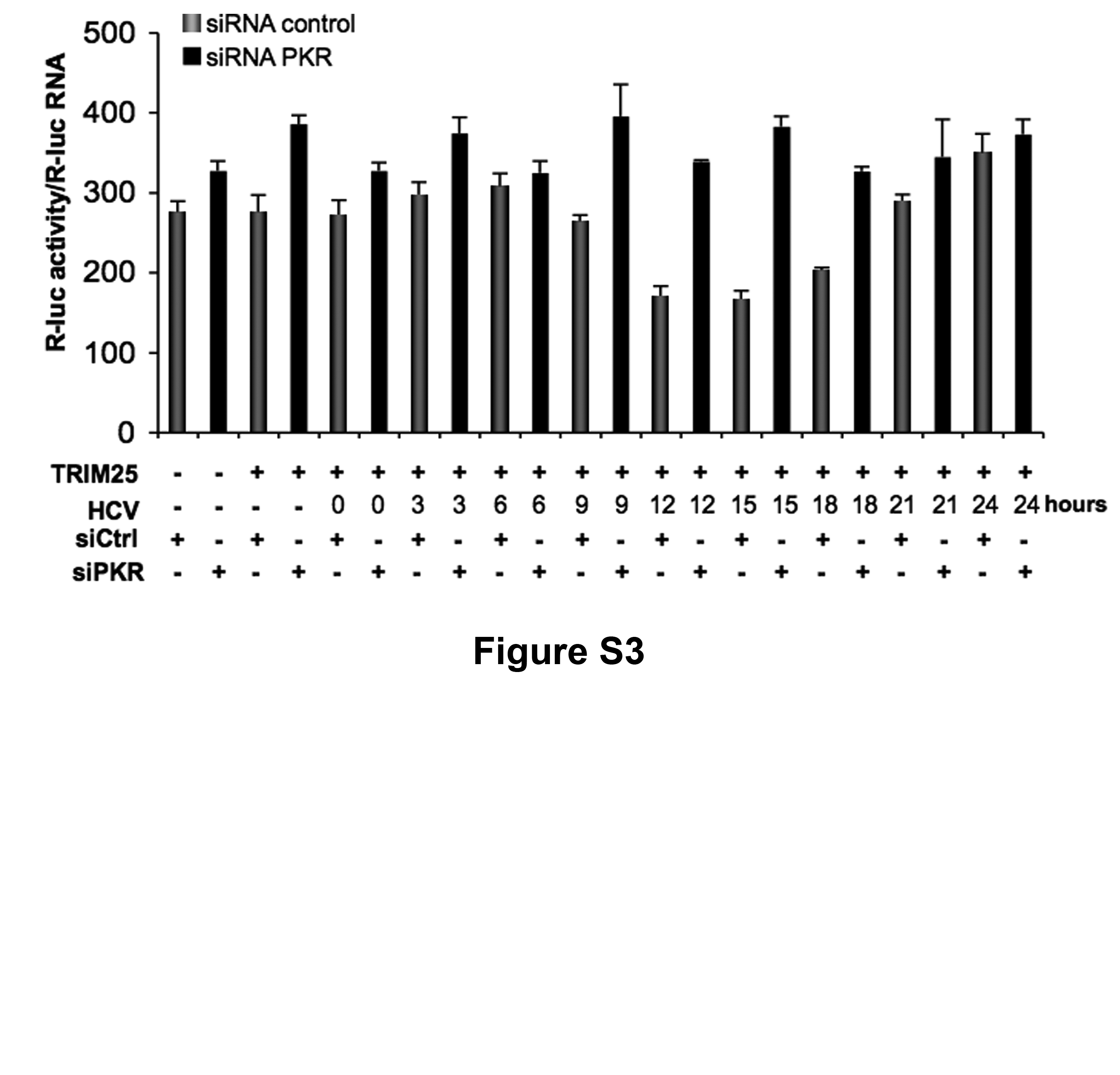

Supplement: Figure S3 — Silencing of endogenous PKR abrogates HCV-mediated inhibition of TK-Renilla luciferase activity. The graphs represent R-luc activity normalized to R-luc RNA in cell extracts corresponding to the experiment described in Figure 7, where Huh7.25.CD81 cells were first transfected with 25 nM of siRNA directed against PKR or with 25 nM of control siRNA and then transfected 24 hrs later with the pGL2-IFNβ-FLUC/pRL-TK-RLUC reporter plasmids and the TRIM25 expressing plasmid. 24 hrs post-transfection, the cells were infected with JFH1 at an m.o.i. of 0.2. Error bars represent the mean ± S.D. for triplicates. (0.71 MB TIF) [file pone.0010575.s003.tif]

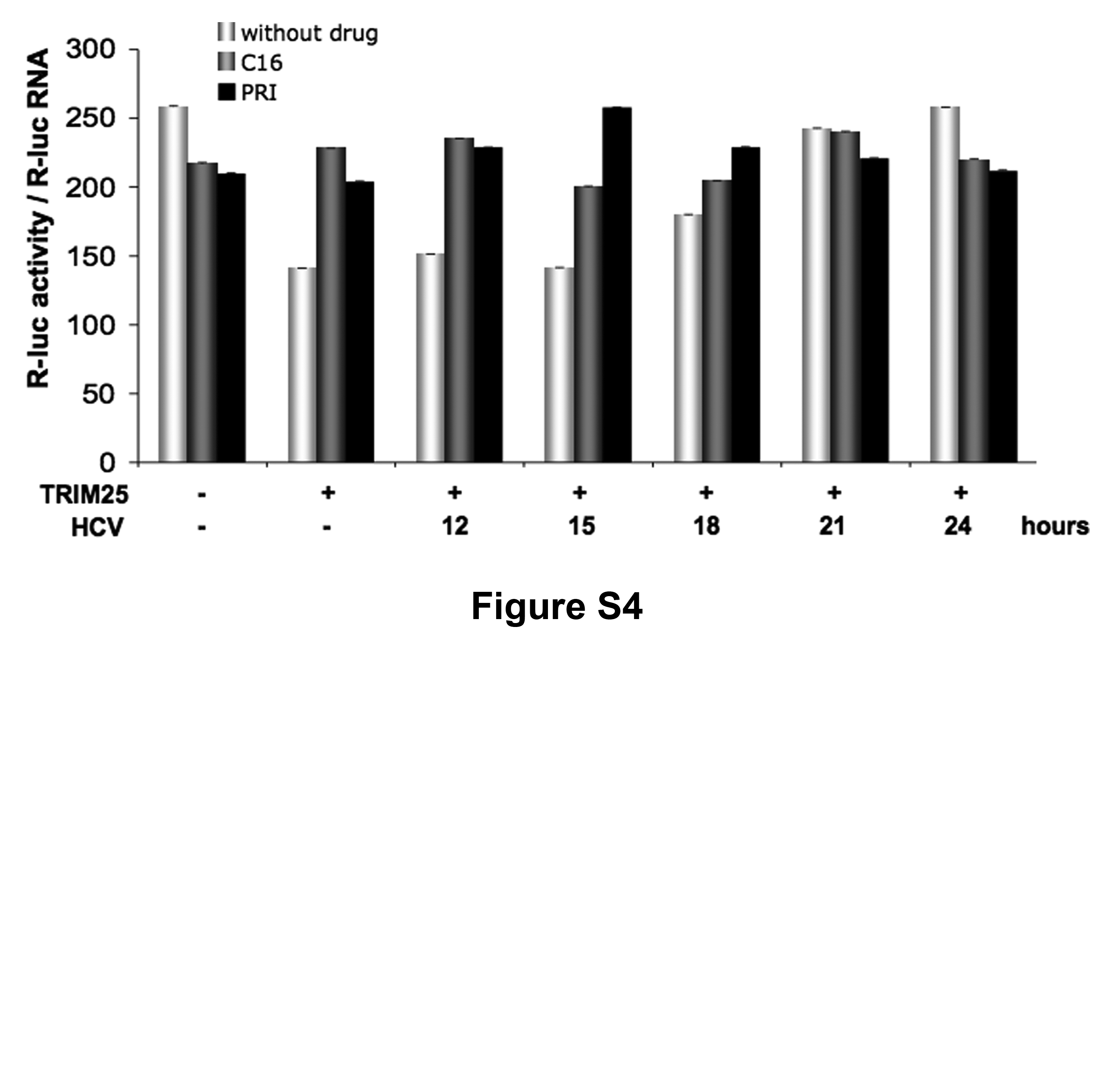

Supplement: Figure S4 — Pharmacological inhibitors of PKR abrogate the HCV-mediated inhibition of TK-Renilla luciferase activity. The graphs represent R-luc activity normalized to R-luc RNA in cell extracts corresponding to the experiment described in Figure 9A, in which Huh7.25.CD81 cells were first transfected with the pGL2-IFNβ-FLUC/pRL-TK-RLUC reporter plasmids and the TRIM25 expressing plasmid. 24 hrs post-transfection, the cells were infected with JFH1 at an m.o.i of 0.2. At 11 hrs post-infection, cells were exposed to 200 µM of C16 or 30 µM of the PRI peptide. Error bars represent the mean ± S.D. for triplicates. (0.99 MB TIF) [file pone.0010575.s004.tif]
